# Influences of resting-state intrinsic functional brain connectivity on the antidepressant treatment response in late-life depression

**DOI:** 10.1017/S0033291722003579

**Published:** 2023-10

**Authors:** Ryan Ahmed, Brian D. Boyd, Damian Elson, Kimberly Albert, Patrick Begnoche, Hakmook Kang, Bennett A. Landman, Sarah M. Szymkowicz, Patricia Andrews, Jennifer Vega, Warren D. Taylor

**Affiliations:** 1 Department of Psychiatry and Behavioral Sciences, Vanderbilt University Medical Center, The Vanderbilt Center for Cognitive Medicine, Nashville, TN, USA; 2 Department of Biostatistics, Vanderbilt University Medical Center, Nashville, TN, USA; 3 Department of Electrical and Computer Engineering, Vanderbilt University, Nashville, TN, USA; 4 Geriatric Research, Education, and Clinical Center, Veterans Affairs Tennessee Valley Health System, Nashville, TN, USA

**Keywords:** Aging, depression, geriatric, fMRI, functional connectivity, antidepressant, treatment, response, outcome

## Abstract

**Background:**

Late-life depression (LLD) is characterized by differences in resting state functional connectivity within and between intrinsic functional networks. This study examined whether clinical improvement to antidepressant medications is associated with pre-randomization functional connectivity in intrinsic brain networks.

**Methods:**

Participants were 95 elders aged 60 years or older with major depressive disorder. After clinical assessments and baseline MRI, participants were randomized to escitalopram or placebo with a two-to-one allocation for 8 weeks. Non-remitting participants subsequently entered an 8-week trial of open-label bupropion. The main clinical outcome was depression severity measured by MADRS. Resting state functional connectivity was measured between *a priori* key seeds in the default mode (DMN), cognitive control, and limbic networks.

**Results:**

In primary analyses of blinded data, lower post-treatment MADRS score was associated with higher resting connectivity between: (a) posterior cingulate cortex (PCC) and left medial prefrontal cortex; (b) PCC and subgenual anterior cingulate cortex (ACC); (c) right medial PFC and subgenual ACC; (d) right orbitofrontal cortex and left hippocampus. Lower post-treatment MADRS was further associated with lower connectivity between: (e) the right orbitofrontal cortex and left amygdala; and (f) left dorsolateral PFC and left dorsal ACC. Secondary analyses associated mood improvement on escitalopram with anterior DMN hub connectivity. Exploratory analyses of the bupropion open-label trial associated improvement with subgenual ACC, frontal, and amygdala connectivity.

**Conclusions:**

Response to antidepressants in LLD is related to connectivity in the DMN, cognitive control and limbic networks. Future work should focus on clinical markers of network connectivity informing prognosis.

**Registration:**

ClinicalTrials.gov NCT02332291

## Introduction

Due to cognitive changes, medical comorbidity, disability, and polypharmacy, treatment of late-life depression (LLD) is inherently complex (Taylor, 2014; Taylor, McQuoid, & Krishnan, 2004). Older depressed adults often do not respond as robustly to antidepressant treatment as do younger adults (Beekman et al., 2002; Tedeschini et al., 2011) and persistent depression is associated with poorer outcomes of medical illness, impaired cognition and dementia, and high rates of suicide (Katon, Unützer, & Russo, 2010; Mulsant, Blumberger, Ismail, Rabheru, & Rapoport, 2014; Nelson, Delucchi, & Schneider, 2013). While clinical, behavioral, and neuropsychological data provide insight on the likelihood of how patients will respond to treatment (Alexopoulos et al., 2005; Nelson et al., 2013; Sheline et al., 2010), such markers are indirect measures of brain function. In contrast, brain-based measures have the potential to disentangle brain network differences that contribute to clinical heterogeneity and variability in the response to treatment (Aizenstein, Khalaf, Walker, & Andreescu, 2014).

Earlier work in LLD examined age-related structural brain changes, often focusing on the hippocampus or white matter hyperintensities (WMH), cerebrovascular-related structural abnormalities common in LLD. Both hippocampal atrophy and greater WMH severity has been associated with poorer antidepressant responses, although these findings are not always consistent across studies (Gunning-Dixon et al., 2010; Hsieh et al., 2002; Sheline et al., 2010; Sneed et al., 2007; Taylor, Aizenstein, & Alexopoulos, 2013a; Taylor, Kudra, Zhao, Steffens, & MacFall, 2014a; Taylor et al., 2014b). A current hypothesis (Taylor et al., 2013a) is that in order for cerebrovascular damage to influence treatment outcomes, WMH would need to disrupt key fiber tracts and impair connectivity between regions of canonical functional networks implicated in depression. These include the default mode network (DMN), a network associated with negativity bias and rumination (Andrews-Hanna, Reidler, Sepulcre, Poulin, & Buckner, 2010; Buckner, Andrews-Hanna, & Schacter, 2008) that often fails to appropriately deactivate in depressed individuals (Sheline et al., 2009) and the cognitive control network (CCN) that is involved in executive function, emotional regulation, and guiding externally directed tasks (Seeley et al., 2007; Zilverstand, Parvaz, & Goldstein, 2017). Past work reports that, compared to normal elderly subjects, depressed elders exhibit altered resting-state functional connectivity across DMN regions (Alexopoulos et al., 2012; Gandelman et al., 2019) and lower functional connectivity within the CCN (Alexopoulos et al., 2012). The limbic network, involved in emotion processing, the emotional response, and memory (Helm et al., 2018), is a third network for consideration. Altered limbic network function, particularly hyperactivity, is associated with greater depression severity (Peluso et al., 2009). Network functional connectivity patterns are dynamic and connectivity patterns in the DMN and CCN change with antidepressant treatment (Karim et al., 2017). It remains unclear whether measures of resting-state functional connectivity pre-treatment can predict antidepressant response in LLD.

Studies examining pre-treatment connectivity as a predictor of response in LLD are sparse and often limited by open-label trial designs or smaller sample sizes. However, they do support that variability in the antidepressant response is associated with functional connectivity differences in the CCN, salience network, reward network, and even sensorimotor regions (Alexopoulos et al., 2012; Andreescu et al., 2013; Karim et al., 2017; Steffens, Wang, & Pearlson, 2019). Randomized clinical trials in midlife major depressive disorder (MDD) support that resting-state connectivity patterns may predict antidepressant treatment response. Higher connectivity between DMN hub regions, specifically the posterior cingulate cortex (PCC) and anterior cingulate cortex (ACC)/medial prefrontal cortex (mPFC) regions, predicted remission to first-line antidepressant regimens (Goldstein-Piekarski et al., 2018). Treatment response is further associated with connectivity differences between the subgenual anterior cingulate cortex (sgACC) and prefrontal regions (Dunlop et al., 2017). The EMBARC study (Trivedi et al., 2016), a multisite, randomized, controlled trial, identified a number of within-network and across-network moderators related to antidepressant response (Chin Fatt et al., 2020). These findings included within-network DMN connectivity and cross-network CCN connectivity, while supporting an important role of limbic network connectivity with the hippocampus emerging as a key region (Chin Fatt et al., 2020; Trivedi et al., 2016).

This study aimed to determine whether regional resting-state functional connectivity measures obtained prior to randomization and treatment were associated with change in depression severity over a randomized, controlled trial. We hypothesized that functional connectivity in the DMN, CCN, and limbic networks would be related to clinical improvement. Based on past work (Alexopoulos et al., 2012; Trivedi et al., 2016), our primary hypotheses were that higher resting within-network connectivity for both the DMN and CCN would be associated with better antidepressant responses. Additionally, given recent work (Chin Fatt et al., 2020; Dunlop et al., 2017), we also tested for select cross-network relationships involving the sgACC and a possible role of the hippocampus. In primary analyses, we focused on change in depression severity during a blinded, controlled trial of escitalopram. In secondary analyses, we tested for moderating effects of regional connectivity on treatment-specific response and change in depression severity over time. In an exploratory aim, we tested for similar relationships during subsequent open-label treatment with bupropion.

## Methods

### Participants

Participants were recruited at Vanderbilt University Medical Center (VUMC; Nashville, TN) through outpatient referrals and response to community advertisements. Enrollment ranged from June 2015 through March 2020.

Criteria for inclusion required subjects be age 60 years or older and meet DSM-IV-TR criteria for MDD with a Montgomery-Asberg Depression Rating Scale (MADRS) (Montgomery & Asberg, 1979) score of 15 or more. Participation required a Mini-Mental State Exam (MMSE) (Folstein, Folstein, & McHugh, 1975) score of 24 or greater with no diagnosis of dementia or other neurological disorder. Exclusion criteria included: (1) other Axis 1 diagnoses, other than anxiety symptoms occurring during depressive episodes; (2) history of substance use disorder in the last 3 years; (3) history of psychosis; (4) acute suicidality; (5) acute grief; (6) MRI contraindications; (7) a failed trial of escitalopram in the current episode; (8) ECT in the last 6 months; and (9) current psychotherapy. Antidepressant medication use at study entry was not an exclusion criterion. After eligibility was confirmed, individuals taking antidepressant medication had those medications tapered and discontinued over several weeks. They were clinically assessed weekly for worsening depression, safety concerns such as emergent suicidality, or development of other adverse events. They could be withdrawn and return to clinical care if these problems developed. Participants were off antidepressant medications for at least two weeks prior to baseline assessments.

All participants provided written informed consent. The VUMC Institutional Review Board approved the study. The study was registered with ClinicalTrials.gov (NCT02332291).

### Assessments

The Mini-International Neuropsychiatric Interview (Sheehan et al., 1998) evaluated psychiatric diagnoses, with findings confirmed by a geriatric psychiatrist. Depression severity was quantified using the MADRS and medical burden was quantified with the Cumulative Illness Rating Scale (CIRS) (Miller et al., 1992). Age of initial depressive episode onset and duration of the current depressive episode was obtained by clinical interview with a geriatric psychiatrist and review of medical records. The MADRS was similarly obtained by a geriatric psychiatrist at each visit.

### Study intervention and clinical visits

Participants were randomized to either escitalopram or placebo in a 2 to 1 allocation. The study statistician (HK) created a sequential predetermined assignment managed by the Vanderbilt Investigational Drug Service to assign participants to each treatment arm. As WMH severity may influence treatment outcomes (Gunning-Dixon et al., 2010; Sheline et al., 2010; Taylor et al., 2013a, 2014a), randomization was stratified by ‘high’ or ‘low’ WMH severity based on a median WMH volume derived from earlier datasets in LLD. The initial cutoff was a WMH volume of 3.86 mL, the median WMH volume observed on 3 T MRI in 145 depressed older adults across prior studies (Chang et al., 2011; Taylor et al., 2014a, 2013b). This stratification threshold was adjusted downward to 2.00mL by the end of the study based on the median WMH volume observed in the current study population. Participants, study physicians, and staff were blinded to treatment allocation.

For phase 1, study medication was started at one tablet daily (either 10 mg of over-encapsulated escitalopram or matching placebo), with the option to increase to two tablets daily as early as week 2. The decision to increase the dose was based on change in depression severity, clinical judgment, tolerability, and patient preference. Participants were assessed every two weeks, by telephone at weeks 2 and 6, and in clinic at weeks 4 and 8.

Participants who could not tolerate study medication or did not remit after 8 weeks had their phase 1 drug tapered over one week before progressing to phase 2, an 8-week open-label trial of bupropion, using the 24-h extended dose formulation. Dosage started at 150 mg daily and increased to 300 mg daily in 2–4 weeks if tolerated. Participants had the option to withdraw if they did not tolerate the 300 mg dose. They could continue on the 300 mg dose or increase to a maximum 450 mg daily as early as week 4 if they tolerated the medication and were not experiencing clinical improvement. Study assessments and depression severity scoring through MADRS followed the same protocol as phase 1.

### MRI acquisition

Participants completed pre-randomization MRI at the Vanderbilt University Institute for Imaging Sciences on a research-dedicated 3.0 T Philips Achieva whole-body scanner (Philips Medical Systems, Best, the Netherlands) using body coil radiofrequency transmission and a 32-channel head coil for reception. Structural imaging included a whole-brain T1-weighted MPRAGE image with TR = 8.75 ms, TE = 4.6 ms, flip angle = 9°, and spatial resolution = 0.89 × 0.89 × 1.2 mm^3^ plus a FLAIR T2-weighted imaging conducted with TR = 10 000 ms, TE = 125 ms, TI = 2700 ms, flip angle = 90°, and spatial resolution = 0.7 × 0.7 × 2.0 mm^3^. Resting-state functional MRI was conducted with eyes open (TR = 2000 ms, echo time = 35 ms, flip angle = 77°, spatial resolution = 2.75 × 2.75 × 3.7 mm^3^, 35 axial slices). WMH volumes were measured on FLAIR images using the Lesion Segmentation Toolbox (Schmidt et al., 2012) as previously described (Gandelman et al., 2019).

### Functional MRI analyses

Resting-state functional images were preprocessed using the CONN toolbox (version 15.g) in SPM12, including realignment of the functional runs and correction for head motion, coregistration of functional and anatomical images for each participant, normalization of the anatomical and functional images to the standard MINI template, and spatial smoothing with a Gaussian filter (6 mm at full width at half maximum). Motion artifacts were further detected by applying the Artifact Detection Toolbox as implemented in CONN. We used a displacement threshold of 0.9 mm and a global signal threshold of *Z* = 5. To effectively mitigate the effects of head motion, denoising in CONN was conducted for white matter (five components extracted) and cerebrospinal fluid (five components extracted) signal, and realignment parameters (Muschelli et al., 2014) with outlier volumes identified by the Artifact Detection Toolbox. We retained all participants with >5 min of scan time after excluding outlier volumes. The resulting blood oxygen level–dependent time series were band-pass filtered (0.01 to 0.1 Hz) to further reduce noise and increase sensitivity.

We selected seed regions of interest (ROIs) for primary seed-to-seed resting state functional connectivity analyses, identified from the original study hypotheses and recent literature (Chin Fatt et al., 2020; Trivedi et al., 2016). These cortical and subcortical ROIs focused on the DMN, CCN, and limbic networks. Using methodology adapted from the EMBARC trial (Chin Fatt et al., 2020; Schaefer et al., 2018), cortical ROI seeds were identified with the Yeo atlas (Yeo et al., 2011). Cortical DMN ROIs included the (1) PCC, (2) mPFC, and (3) rostral/pregenual ACC (rACC). Cortical CCN ROIs included the (4) dorsal ACC (dACC) and (5) dorsolateral prefrontal cortex (DLPFC), while the limbic network included the (6) sgACC and (7) orbitofrontal cortex (OFC). Subcortical ROIs were identified using the WakeForest Anatomical Atlas and included the (8) anterior hippocampus and the (9) amygdala. Aside from the PCC and sgACC, where the seed crossed midline, other regions were measured bilaterally in separate ROIs (refer to online Supplementary Table S1 for full ROI details). Following *a priori* hypotheses, we generated seed-to-seed pairs for evaluation of functional connectivity (refer to online Supplementary Table S2 for all seed-to-seed pairs examined) and extracted individual-level beta values for each ROI pair of interest.

To account for individual differences in gray matter volume within each ROI, each subject was processed with FreeSurfer7 using the standard recon-all procedure. As outlined by the Yeo group (https://bit.ly/3wv0rZo), the Schaefer parcellations were projected to each subject's surface using the FreeSurfer procedure ‘mri_surf2surf’ and then transferred to labels using ‘mri_aparc2seg’. These labels were then used to calculate the volume of each ROI with ‘mri_segstats.’

### Statistical analyses

Statistical analyses were conducted in R Statistical Software (version 4.0.3, https://cran.r-project.org). Summary statistics were used to characterize the participants.

First, we sought to determine what functional connectivity pairs were associated with post-treatment depression severity. These primary analyses of the initial blinded phase examined the relationship between pre-randomization resting functional connectivity and clinical improvement assessed by the final assessed MADRS score. We selected final MADRS score as the primary outcome over categorical characterizations such as remission or response to preserve power given the relatively small number of individuals assigned to placebo who achieved those thresholds. We created a general linear model predicting final MADRS score, including all pairwise seed-to-seed connectivity measures and key covariates (baseline MADRS score, treatment assignment, time in the study, age, gender, and WMH volume). Using this approach, we had no missing data for individuals with usable fMRI data. Backward stepwise elimination was used to determine which seed-to-seed connectivity measures were most strongly associated with final MADRS score. Using the step() function implemented in R Statistical Software, an initial linear mixed model with all ROI pairs and covariates was specified. Key covariates were retained in the final model and fixed ROI effects were dropped iteratively based on improvement of Akaike information criterion (AIC) value until either (1) subsequent models no longer improved AIC or (2) a single independent variable of interest remained. All connectivity pairs and total WMH volume were scaled using variable means and standard deviations to keep all predictors comparable during backward elimination.

To account for potential regional volumetric differences that would affect study results, a composite gray matter volume measure was then added as a covariate to the final backwards elimination model. This measure derived from a principal component analysis (PCA) that accounted for composite gray matter across all regions included within that final backward elimination model. A single principal component was estimated from standardized brain region gray matter volumes using varimax rotation, and component scores were extracted as a covariate.

Secondary analyses examined whether pre-randomization resting connectivity measures were associated with treatment-specific changes in the trajectory of depression severity change over time. This approach used longitudinal mixed effects models examining MADRS score as a repeated measure and independent variables of seed connectivity, treatment assignment, and time, controlling for covariates of age, gender, and WMH volume. Initial models tested for a three-way statistical interaction between connectivity, treatment, and time. When that interaction term did not achieve statistical significance at the false positive rate less than 0.05, we removed the three-way interaction term and examined interactive effects between seed connectivity and time. These models also included a treatment by time interaction, but that was not the focus of analyses. These secondary analyses were considered as exploratory. The sample was not sufficiently powered to detect differences in the relationship between resting functional connectivity and clinical course between the treatment arms, particularly given the unequal randomization between arms. However, these would be useful hypothesis-generating data. For these reasons we did not adjust for multiple comparisons.

Finally, we conducted exploratory analyses of the subsample of individuals who progressed to the subsequent open-label bupropion phase. Due to the smaller sample size, we did not pursue the backwards elimination approach but rather used similar approaches as in the secondary analyses of the blinded trial. These analyses did not include treatment assignment as a dependent variable as all were on the same treatment.

To account for regional volumetric differences in these secondary and exploratory analyses, gray matter was added as a covariate to each mixed model with statistically significant findings. Standardized gray matter volumes (i.e. (raw regional gray matter volume – mean regional gray matter volume)/standard deviation of regional gray matter volume) from each region of the ROI-to-ROI pair were included as a covariate in final models.

These secondary and exploratory analyses were affected by missing outcome data. Time points which did not have an outcome measurement (i.e. missing total MADRS score) were excluded via listwise deletion, but subjects with outcome data at any time point were included. Mixed effect modeling accounted for missing data by calculating a maximum likelihood estimate, which produced an unbiased parameter estimate since data met the assumption of being either missing at random or missing completely at random.

## Results

**Fig. 1.**
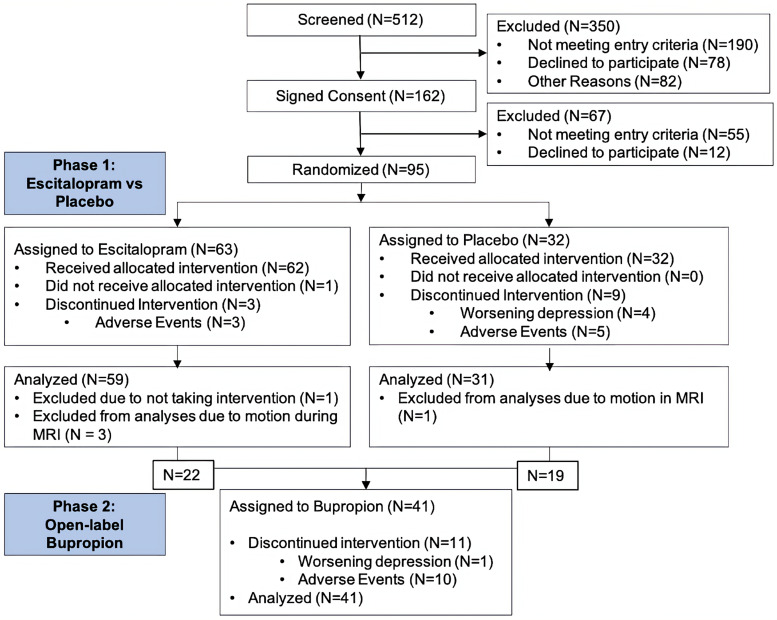
Consort diagram. The majority of people who were not eligible at the screening visit were due to MRI contraindications, not meeting depression severity criterion, or having other comorbid neurological or psychiatric disease. Most placebo arm withdrawals were for worsening symptoms. Most drug arm withdrawals were for medication intolerance.

The study enrolled 162 depressed elders (Fig. 1), with 95 individuals completing baseline procedures and subsequent randomization. The majority of withdrawn individuals were excluded due to concerns for MRI safety based on prior surgeries or medical procedures identified after obtaining initial informed consent and before progressing to the baseline visit. Although study eligibility allowed for a MMSE score of 24 or greater, all randomized participants exhibited a score of 26 or greater, so could be considered as cognitively intact. There were no significant treatment group differences in baseline demographic data or dose equivalents (Table 1), only a treatment effect where the escitalopram cohort had significantly lower final MADRS scores. Overall, the population exhibited depression chronicity, with the mean duration of the current depressive episode approaching three years (range 15–5141 days).

**Table 1.** Demographics table

	Placebo (*N* = 31)	Drug (*N* = 59)	Test value	*p* value
Age, years	66.29 (4.82)	65.92 (4.42)	*t* = 0.36	0.72
Sex, % female (*N*)	61.29% (19)	55.93% (33)	χ^2^ = 0.24	0.62
Education	15.32 (1.99)	15.34 (2.31)	*t* = −0.04	0.97
MMSE	29.19 (1.22)	29.46 (0.84)	*t* = −1.08	0.29
CIRS	6.16 (3.82)	5.44 (3.34)	*t* = 0.89	0.38
WMH volume (ml)	2.77 (3.20)	2.53 (3.83)	*t* = 0.30	0.76
Age of initial onset (y)	32.7 (18.70)	32.4 (19.2)	*t* = 0.09	0.93
Duration of current episode (days)	1085.0 (1136.5)	1038.4 (956.2)	*t* = 0.21	0.84
Baseline MADRS	25.84 (6.38)	26.32 (5.10)	*t* = −0.36	0.72
Final MADRS	19.52 (11.82)	12.41 (10.35)	*t* = 2.83	0.01
Final dose (mg)	18.00 (4.07)	17.37 (4.44)	*t* = 0.67	0.51

CIRS, Cumulative Illness Rating Scale; MADRS, Montgomery-Asberg Depression Rating Scale; MMSE, Mini-Mental State Exam; WMH, white matter hyperintensities (in milliliters).

Continuous variables presented as mean (standard deviation), with categorical variables presented as percent (N). Continuous variables were compared between treatment arms using pooled, two-tailed tests with 88 degrees of freedom. Categorical variables were compared using a χ^2^ test with 1 degree of freedom.

Of the 95 participants who were randomized (Fig. 1), one participant withdrew from the study after randomization but before receiving study drug. Four participants were excluded from analyses due to motion during MRI. Of the remaining 90 participants, 59 received escitalopram and 31 received placebo. Three individuals randomized to escitalopram and 9 individuals randomized to placebo withdrew early from the blinded phase due to worsening depression or poor tolerability, with the remainder completing the blinded phase. Forty-one participants (22 from the escitalopram arm and 19 from the placebo arm) subsequently entered the open-label bupropion phase, including 2 individuals in the escitalopram arm and 4 individuals in the placebo arm who withdrew early from the blinded phase. Eleven of these individuals withdrew early and the remaining 30 participants completed the open-label phase.

### Primary analyses predicting final MADRS score

We integrated all *a priori* regional resting-state functional connectivity pairs (online Supplementary Table S1) into a single model. After completing backwards elimination and adding the gray matter covariate to the model, the final model identified six regional resting functional connectivity pairs that were significantly associated with final MADRS score (Table 2). Regions in the CCN (left DLPFC – left dorsal ACC) and in the limbic network (right OFC – left amygdala) exhibited a positive relationship, with greater functional connectivity being associated with a higher final MADRS score. Regions in the DMN (PCC – left mPFC, PCC –sgACC, and right mPFC – sgACC) and the limbic network (right OFC – left hippocampus), exhibited a negative relationship, where greater functional connectivity was associated with lower final MADRS score.

**Table 2.** Functional connectivity pairs associated with final depression severity in initial blinded trail

	Network	Estimate	s.e.	*t*-value	*p* value
Retained demographics/Covariates					
Age		−0.27	0.18	−1.50	0.1381
Gender		−2.33	1.85	−1.26	0.2104
Baseline MADRS		0.90	0.15	5.98	<0.0001
Treatment assignment		−4.03	1.66	−2.43	0.0175
Time in study		−1.72	0.40	−4.25	<0.0001
WML volume (total)		0.0002	0.0002	0.97	0.3328
Gray matter (Principal component)		−3.32	0.94	−3.53	0.0007
Significant positive ROI Pairs					
DLPFC (left) –dACC (left)	CCN	5.23	1.36	3.84	0.0003
OFC (right) – Amygdala (left)	Limbic	2.88	0.86	3.34	0.0013
Significant negative ROI Pairs					
DLPFC (left) – dACC (right)	CCN	−2.34	1.05	−2.23	0.0288
PCC – mPFC (left)	DMN	−1.75	0.87	−2.01	0.0485
PCC – sgACC	DMN	−3.85	1.20	−3.21	0.0020
mPFC (right) – sgACC	DMN	−2.61	0.88	−2.96	0.0041
OFC (right) – HPC (left)	Limbic	−4.28	1.07	−4.00	0.0001
ROI Pairs retained in the final model that did not reach statistical significance					
PCC – rACC (right)	DMN	1.18	0.88	1.34	0.1855
OFC (left) – HPC (left)	Limbic	1.59	0.89	1.79	0.0780
OFC (left) – Amygdala (right)	Limbic	1.49	0.96	1.55	0.1248

MADRS, Montgomery-Asberg Depression Rating Scale; DLPFC, dorsolateral prefrontal cortex; dACC, dorsal anterior cingulate cortex; CCN, cognitive control network; OFC, orbitofrontal cortex; PCC, posterior cingulate cortex (bilateral); mPFC, medial prefrontal cortex; DMN, default mode network; rACC, rostral anterior cingulate cortex; sgACC, subgenual anterior cingulate cortex (bilateral).

In this general linear model examining data from the blinded trial of escitalopram and placebo, the outcome variable was final MADRS score. A positive relationship indicated that higher functional connectivity between ROI seeds was associated with a higher final MADRS score. A negative relationship indicated that higher functional connectivity between ROI seeds was associated with a lower final MADRS score.

### Secondary analyses examining treatment and time effects

**Fig. 2.**
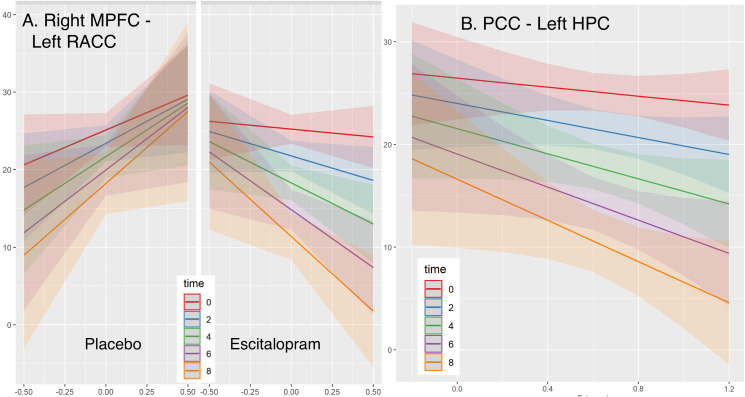
Secondary analyses of change in depression severity based on functional connectivity patterns. (*a*) Initial secondary analyses, after adjusting for regional gray matter volumes, tested for a moderating effect of seed-to-seed FC on the change in depression severity in response to treatment assignment (Time by treatment by FC interaction term; *t* = −2.38, df = 326, *p* = 0.0184). Higher FC between the right mPFC and left rACC (DMN) is associated with better clinical outcomes for individuals assigned to escitalopram, but worse outcomes for those assigned to placebo. (*b*) After removing the three-way interaction term, after adjusting for regional gray matter volumes, higher FC between the PCC and left hippocampus was associated with better clinical response over time (Time by treatment interaction term, *t* = −2.07, df = 327, *p* = 0.0388). No other seed-to-seed FC measure exhibited statistically significant three-way or treatment by time interaction terms. FC, functional connectivity; mPFC, medial prefrontal cortex; rACC, rostral anterior cingulate cortex; PCC, posterior cingulate cortex; *x*-axis = connectivity beta values; *y*-axis = MADRS score (0 to 60 scale). Time displayed in weeks.

We observed a single significant three-way interaction between resting-state connectivity, treatment, and time (full statistical details in online Supplementary Table S2). Greater connectivity between the right mPFC and left rACC was associated with lower MADRS scores over time in the escitalopram arm, but less change in MADRS score over time for those allocated to placebo (*t* = −2.37, 326df, *p* = 0.0184; Fig. 2a).

After removing the three-way interaction term, we tested for an interactive effect between resting functional connectivity and time predicting MADRS score. We observed a significant interaction between PCC – left hippocampal connectivity and time (*t* = −2.07, 327df, *p* = 0.0388; Fig. 2*b*), where increased connectivity was associated with a greater decrease in MADRS score over time.

### Exploratory analysis of subsequent open-label bupropion trial

In the open-label bupropion trial we observed isolated interactive effects between pre-randomization resting state functional connectivity and time on MADRS score. Within the DMN, higher connectivity between the sgACC and left mPFC was associated with lower MADRS scores over time (*t* = −2.16, 124, df, *p* = 0.0324). In parallel, connectivity between the right OFC and right amygdala was associated with higher MADRS scores over time (*t* = 1.53, 124, df, *p* = 0.0149).

## Discussion

In this single-site, two-phase randomized, controlled antidepressant trial in LLD, pre-treatment resting-state functional connectivity in DMN, limbic, and CCN regions significantly predicted clinical outcomes. Beyond primary analyses associating pre-treatment regional resting connectivity measures in these networks with post-treatment depression severity, secondary analyses suggested that resting connectivity within the DMN differentially moderated response to treatment assignment and was associated with change in depression severity over time (Fig. 2). Exploratory analyses of individuals who did not respond to initial treatment and progressed to the second open-label study phase associated change in depression severity with sgACC resting state functional connectivity.

These findings are largely concordant with past work in younger adult cohorts associating pre-treatment DMN, sgACC, and hippocampal resting functional connectivity patterns with response to antidepressant medications (Chin Fatt et al., 2020; Dunlop, Talishinsky, & Liston, 2019). Past work suggests that higher resting connectivity between anterior and posterior nodes of the DMN predicts better pharmacotherapy response (Andreescu et al., 2013; Dunlop et al., 2019; Goldstein-Piekarski et al., 2018),a finding replicated in our primary analysis. Data from the EMBARC trial expanded these results, associating higher within-network DMN functional connectivity more broadly with better response to sertraline over placebo (Chin Fatt et al., 2020).

In primary analyses, higher pre-treatment resting connectivity between the sgACC with both anterior and posterior DMN hubs was also associated with lower post-trial depression severity (Table 2) and sgACC connectivity with additional regions was associated with change in depression severity over the subsequent open-label trial. Substantial work associates the response to antidepressant medications and cognitive behavioral therapy with both sgACC activity (Konarski et al., 2009; Mayberg et al., 1997) and sgACC functional connectivity (Dunlop et al., 2017, 2019; Kozel et al., 2011). Our findings associating higher sgACC resting connectivity with better treatment responses are concordant with this literature, extending those findings into older adults. Analyses of the subsequent open-label bupropion trial further suggest that broader differences in sgACC connectivity may be seen in individuals who did not respond to either study trial. Given the study design, such individuals cannot clearly be described as being treatment resistant, however they may potentially benefit from pharmacological augmentation, neuromodulation or rapidly-acting antidepressants such as ketamine (Baeken, Duprat, Wu, De Raedt, & van Heeringen, 2017; Nakamura et al., 2021).

Just as the hippocampus emerged as a key hub predicting antidepressant response in the EMBARC study (Chin Fatt et al., 2020), our findings also highlight the hippocampus. Involvement of the hippocampus may be particularly salient in LLD given past work associating LLD with smaller hippocampal volumes and hippocampal atrophy (Hsieh et al., 2002; Taylor et al., 2014b). The bilateral hippocampi are integral components of the DMN (Greicius, Supekar, Menon, & Dougherty, 2009) and connectivity between the hippocampus and DMN regions such as the PCC may have functional consequences, such as contributing to episodic memory deficits (Bai et al., 2009; Schott et al., 2013; Sestieri, Corbetta, Romani, & Shulman, 2011). Such cognitive deficits are in turn associated with poor antidepressant response (Sheline et al., 2010). Intriguingly, we previously associated a poorer antidepressant response with WMH damage to the posterior limb of the cingulum bundle (Taylor et al., 2014a), the fiber tract serving as the structural connection between the anterior hippocampus and PCC.

We previously reported volumetric differences in the OFC in LLD (Taylor et al., 2007). In this study we observed a differential effect of OFC resting functional connectivity. Higher resting connectivity with the hippocampus was associated with lower final MADRS scores, but conversely higher connectivity with the amygdala was associated with higher final MADRS scores. Limbic regions are associated both with depression (Bremner, Fani, Cheema, Ashraf, & Vaccarino, 2019; Siegle, Steinhauer, Thase, Stenger, & Carter, 2002) and with the physiologic response to stress (Rajmohan & Mohandas, 2007). As the limbic network has reciprocal excitatory and inhibitory projections (Radley, 2012), it is possible that greater connectivity with the hippocampus may facilitate hippocampal efforts to regulate that stress response (Herman et al., 2003). In contrast, higher OFC connectivity with the amygdala may challenge stress or emotional regulation, contributing to both depression and potentially decreasing the likelihood of a treatment response. This finding deserves further study, as past studies have associated a better response to antidepressant medications with higher amygdala functional connectivity with frontocingulate regions (Klimes-Dougan et al., 2018; Vai et al., 2016). This reflects broader issues in the field about challenges in understanding inconsistencies in findings across functional neuroimaging studies.

Finally, higher resting-state connectivity within the CCN was associated with poorer clinical response, or conversely, lower within-network CCN connectivity was associated with better response. This is surprising given previous findings in LLD demonstrating that lower CCN connectivity is related to persistent depression, executive dysfunction, and poor antidepressant medication response (Alexopoulos et al., 2012). Our finding may reflect heterogeneity in the LLD population. Some past work (Alexopoulos et al., 2012) has focused on executive dysfunction, which may enrich samples for CCN dysfunction. In contrast, our sample exhibited intact cognitive performance at screening and poorer performance on executive function tests were not a requirement for study entry.

Exploratory analyses examining outcomes from the open-label bupropion trial should be viewed cautiously. Sample size and multiple comparisons are an issue with these analyses, as less than half of study participants progressed to that study phase. Moreover, by definition, this approach eliminated individuals with a more ‘favorable’ network connectivity pattern who responded during the blinded trial.

A strength of this study included its rigorous clinical design as a blinded, controlled trial. However, limitations include a modest overall sample size, with the allocation resulting in a small placebo arm. The number of subjects excluded because of past surgical history due to concerns for MRI safety may have reduced study generalizability to more medical ill elders. Moreover, while antidepressant trial durations of 8 weeks are common, some individuals may need 12 weeks or longer to exhibit a clinical response. Thus our design may have classified some individuals who needed more time on medication as ‘nonresponders’. Multiple comparisons are an additional limitation in our secondary and exploratory analyses. In order to reduce the number of total comparisons, we tested a set number of *a priori* seed-to-seed regions. This approach negates the ability to identify connectivity patterns related to treatment response that involve regions outside our *a priori* seeds. However, the study was not powered to detect differences in the relationship between allocation groups in connectivity measures and clinical change. Moreover, our findings would not have survived statistical correction for multiple comparisons. Thus, even though our results are generally concordant with past work, they should be viewed cautiously as hypothesis-generating findings. Additionally, while the use of backwards elimination for primary analyses allowed a focus on a single rather than multiple models, it does carry the limitation that variables removed early in the process are not reintroduced, even if they would have been statistically significant in the final model (Chowdhury & Turin, 2020). Finally, as connectivity patterns change during antidepressant treatment (Karim et al., 2017), obtaining only a pre-treatment MRI precluded us from examining changes in connectivity patterns over time that may be related to recovery.

In conclusion, pre-treatment resting state functional connectivity patterns across multiple intrinsic networks are associated with the response to pharmacotherapy in older depressed adults. This advances our understanding of the neurobiological profile that characterizes an individual who will likely respond to first-line antidepressant treatment and extends it into older adults. When combined with previous work in this area (Chin Fatt et al., 2020; Gandelman et al., 2019; Goldstein-Piekarski et al., 2018; Karim et al., 2017), our findings support that network connectivity patterns may serve as proximal identifiers of favorable response to antidepressant treatment in complex patient populations. Given the single-site nature of the study and relatively small sample size for a clinical trial, future research should work to both replicate these observations and translate these findings into accessible clinical markers. This could allow for clinical stratification of patients into those likely to have a good response to first- or second-line pharmacotherapy, or inform the identification of a treatment-resistant phenotype who may benefit from earlier intervention with pharmacological augmentation or neuromodulation.

## Supplementary material

For supplementary material accompanying this paper visit https://doi.org/10.1017/S0033291722003579. click here to view supplementary material
