# Supplementary material for: Influences of resting-state intrinsic functional brain connectivity on the antidepressant treatment response in late-life depression
Source: Psychol Med. 2022 Dec 9;53(13):6261–70. doi: 10.1017/S0033291722003579 (PMC10250562; doi:10.1017/S0033291722003579)
Supplement: Supplementary file 1 [file S0033291722003579sup001.pdf]

SUPPLEMENTAL MATERIALS:

Influences of Intrinsic Patterns of Functional Brain Connectivity on the Antidepressant Treatment Response in Late-Life Depression

Supplemental Table 1 – Cortical Regions of Interest (ROIs) and Composite Seeds Chosen for Functional Connectivity Analysis

ROI	*Network*	*Area (Schaefer)**	*Parcel (Schaefer)†*
Posterior Cingulate Cortex (PCC)/ Precuneus	DMN	LH/pCunPCC_3	192
	DMN	RH/pCunPCC_4	395
**Consolidated Bilateral PCC**	**DMN**		**Combined 192 and 395**
**Rostral/Pregenual Anterior Cingulate Cortex (rACC)**	**DMN**	**LH/PFC_9**	**174**
	**DMN**	**RH/PFCdPFCm_3**	**381**
Medial Prefrontal Cortex (mPFC)	DMN	LH/PFC_3	168
	DMN	LH/PFC_8	173
**Consolidated Left mPFC**	**DMN**		**Combined 168 and 173**
	DMN	RH/PFCdPFCm_1	379
	DMN	RH/PFCdPFCm_2	380
	DMN	RH/PFCdPFCm_4	382
**Consolidated Right mPFC**	**DMN**		**Combined 379, 380, and 382**
Subgenual Anterior Cingulate Cortex (sgACC)‡	Limbic Network	LH/OFC_7	177‡
	DMN	RH/PFCdPFCm_1	568‡
**Consolidated Bilateral sgACC**	**DMN**		**Combined 177‡ and 568‡**
Orbitofrontal Cortex (OFC)	Limbic Network	LH/OFC_1	114
	Limbic Network	LH/OFC_3	116
	Limbic Network	LH/OFC_4	117
**Consolidated Left OFC**	**Limbic Network**		**Combined 114, 116, and 117**
	Limbic Network	RH/OFC_1	319
	Limbic Network	RH/OFC_3	321
	Limbic Network	RH/OFC_5	323

**Consolidated Right OFC**	**Limbic Network**		**Combined 319, 321, 323**
Dorsolateral Prefrontal Cortex (DLPFC)	CCN	LH/PFCl_5	139
	CCN	LH/PFCl_6	140
	CCN	LH/PFCl_8	142
**Combined Left DLPFC**	**CCN**		**Combined 139, 140, and 142**
	CCN	RH/PFCl_9	349
	CCN	RH/PFCl_11	351
**Combined Right DLPFC**	**CCN**		**Combined 349 and 351**
**Dorsal Anterior Cingulate Cortex (dACC)**	**CCN**	**LH/Cing_1**	**146**
	**CCN**	**RH/Cing_2**	**359**

Regions of interest were chosen based on current fMRI literature in late-life depression (LLD). Using the functional connectivity based Schaefer parcellation of the Yeo atlas, specific parcels were chosen to represent ROIs. Final analytic seeds used for functional connectivity analysis are bolded. * = Area is described using the notation directly from the Schaefer parcellation. † = parcel number is derived from the 400-parcel, 7-network Schaefer atlas unless otherwise noted. ‡ = for the sgACC, parcels were chosen from the 600-parcel, 7-network Schaefer atlas. ROI = region of interest; DMN = default mode network; LH = left hemisphere; RH = right hemisphere; CCN = cognitive control network.

Supplemental Table 2 – Seed-to-Seed Pair Results for Secondary Analyses

**Three-Way Interaction Treatment * Time * Functional Connectivity**					
DMN Primary Seeds					
Seed 1	*Seed 2*	*Estimate*	*SE*	*T-value*	*P-value*
PCC	mPFC_L	1.79	1.49	1.20	0.2306
	mPFC_R	0.30	1.71	0.17	0.8633
	rACC_L	0.24	1.63	0.15	0.882
	rACC_R	-1.69	1.58	-1.07	0.285
	sgACC	0.06	0.92	0.07	0.9449
	hpc_L	-0.08	1.04	-0.08	0.9356
	hpc_R	-1.15	1.28	-0.90	0.3671
mPFC_L	rACC_L	-0.05	1.60	-0.03	0.9738
	rACC_R	1.03	1.42	0.73	0.467
	sgACC_B	-2.60	1.54	-1.68	0.0932
**mPFC_R**	**rACC_L**	**-3.35**	**1.41**	**-2.38**	**0.0179**
	rACC_R	-2.04	1.59	-1.28	0.2006
	sgACC	-1.09	1.38	-0.79	0.4291
Limbic Network Primary Seeds					
Seed 1	*Seed 2*	*Estimate*	*SE*	*T-value*	*P-value*
OFC_L	hpc_L	-1.27	1.40	-0.91	0.3629
	hpc_R	-0.65	1.58	-0.41	0.682
	amyg_L	0.83	1.58	0.53	0.5988
	amyg_R	-0.09	1.46	-0.06	0.9515
OFC_R	hpc_L	-1.95	1.33	-1.47	0.1433
	hpc_R	-0.18	1.56	-0.11	0.9088
	amyg_L	-0.61	1.82	-0.34	0.737
	amyg_R	-0.18	1.02	-0.17	0.8613
sgACC_B	hpc_L	0.77	0.98	0.78	0.4341
	hpc_R	1.32	1.18	1.12	0.265
	amyg_L	0.66	1.22	0.54	0.5862
	amyg_R	-0.59	1.26	-0.47	0.6372
CCN Primary Seeds					
Seed 1	*Seed 2*	*Estimate*	*SE*	*T-value*	*P-value*
DLPFC_L	dACC_L	0.19	1.09	0.18	0.8583
	dACC_R	-0.49	1.08	-0.45	0.6544
DLPFC_R	dACC_L	-1.40	1.28	-1.09	0.2771
	dACC_R	0.44	1.42	0.31	0.757
**Two-Way Interaction Time * Functional Connectivity**					
DMN Primary Seeds					
Seed 1	*Seed 2*	*Estimate*	*SE*	*T-value*	*P-value*
**PCC**	mPFC_L	-0.17	0.71	-0.24	0.8085
	mPFC_R	0.25	0.80	0.32	0.7507

	rACC_L	0.72	0.81	0.89	0.3741
	rACC_R	0.70	0.79	0.89	0.3736
	sgACC	-0.76	0.44	-1.73	0.0852
	**hpc_L**	**-0.98**	**0.47**	**-2.09**	**0.0372**
	hpc_R	-0.47	0.60	-0.79	0.4325
mPFC_L	rACC_L	0.01	0.73	0.02	0.9857
	rACC_R	0.32	0.69	0.46	0.6470
	sgACC	-0.47	0.73	-0.64	0.5201
mPFC_R	rACC_L	-1.14	0.66	-1.72	0.0868
	rACC_R	-0.39	0.71	-0.55	0.5810
	sgACC	0.03	0.65	0.05	0.9589
Limbic Network Primary Seeds					
Seed 1	*Seed 2*	*Estimate*	*SE*	*T-value*	*P-value*
OFC_L	hpc_L	-0.29	0.65	-0.44	0.6567
	hpc_R	0.60	0.78	0.76	0.4473
	amyg_L	1.08	0.75	1.44	0.1509
	amyg_R	0.06	0.72	0.09	0.9318
OFC_R	hpc_L	-1.09	0.66	-1.65	0.0992
	hpc_R	0.32	0.76	0.42	0.6718
	amyg_L	1.54	0.82	1.89	0.0603
	amyg_R	-0.06	0.48	-0.13	0.8968
sgACC	hpc_L	-0.13	0.47	-0.27	0.7866
	hpc_R	-0.61	0.56	-1.09	0.2767
	amyg_L	-0.89	0.52	-1.72	0.0857
	amyg_R	-0.45	0.62	-0.73	0.4641
CCN Primary Seeds					
Seed 1	*Seed 2*	*Estimate*	*SE*	*T-value*	*P-value*
DLPFC_L	dACC_L	-0.24	0.53	-0.46	0.6474
	dACC_R	-0.79	0.50	-1.57	0.1178
DLPFC_R	dACC_L	-0.41	0.62	-0.67	0.5054
	dACC_R	-0.04	0.68	-0.07	0.9477

Initial models examined FC for each seed pair in 3-way interactions (treatment assignment by time by functional connectivity), including covariates of age and sex. When those were not statistically significant, the 3-way interaction term was removed and the remaining two-way interactions were examined (treatment assignment by functional connectivity; time by functional connectivity). The table above shows statistical analyses of each seed-to-seed pair, arranged by the network represented by seed 1 in the pair. Statistically significant interactions between pairs ($p<0.05$) are denoted with bolding and an asterisk.

Note that for significant statistical interactions, as a final step gray matter volumes of the seed regions were added to the final model. This statistical approach and final statistical result data re included in the main manuscript.

Tx = treatment assignment; Ti = time; FC = functional connectivity; DMN = default mode network; _L = left hemispheric seed; _R = right hemispheric seed; PCC = posterior cingulate cortex; mPFC = medial prefrontal cortex; rACC = rostral anterior cingulate cortex; sgACC =

subgenual anterior cingulate cortex; hpc = hippocampus; OFC = orbitofrontal cortex; amyg = amygdala; CCN = cognitive control network; DLPFC = dorsolateral prefrontal cortex; dACC = dorsal anterior cingulate cortex.
